# Corneal epithelial and stromal thickness changes in myopic orthokeratology and their relationship with refractive change

**DOI:** 10.1371/journal.pone.0203652

**Published:** 2018-09-25

**Authors:** Wook Kyum Kim, Bong Jun Kim, IK-Hee Ryu, Jin Kook Kim, Sun Woong Kim

**Affiliations:** 1 B & VIIT Eye Center, Seoul, South Korea; 2 Department of Ophthalmology, Yonsei University Wonju College of Medicine, Wonju, Gangwon-do, South Korea; Xiamen University, CHINA

## Abstract

**Purpose:**

To investigate topographic changes in corneal epithelial thickness (CET) and stromal thickness following orthokeratology (OK) and to determine associated factors affecting refractive changes.

**Methods:**

This study investigated the topographic changes in CET and stromal thickness in 60 myopic eyes that were fitted with OK lenses. CET and stromal thickness were obtained using spectral-domain optical coherence tomography (OCT) before and after OK lens wear. Changes in refractive error and corneal topography data were obtained. The correlation between refractive change and corneal thickness change, and various refractive, lens, and topographic parameters were analyzed using simple regression analysis.

**Results:**

Mean refractive error changed by 1.75 ± 0.79 diopters (D). The mean CET of the center zone (2 mm in diameter), paracenter (2 to 5 mm annular ring: 1 to 2.5 mm from center), and mid-periphery (5 to 6 mm annular ring: 2.5 to 3 mm from center) changed by -8.4, -1.4, and +2.7 μm, respectively, after OK lens wear. There was an increase of 2.0, 3.3, and 3.9 μm, respectively, in the center, paracenter, and mid-periphery of the stroma. A larger refractive correction was associated with a flatter base curve of the lens, larger decrease in the central epithelium, and smaller treatment diameter in corneal topography.

**Conclusion:**

OK lenses caused the central corneal epithelium to thin while the mid-peripheral epithelium and stroma became thicker. Refractive changes during OK are associated with changes in central epithelial thickness, while stromal changes did not contribute significantly.

## Introduction

Orthokeratology (OK) is used to reduce the refractive error in myopic subjects through the use of reverse-geometry rigid gas-permeable lenses, which are fitted with a base curve flatter than the central corneal curvature [1, 2]. Numerous studies have reported clinical effectiveness for the correction of mild-to-moderate myopia using the overnight wear modality. Furthermore, recent publications have shown an inhibitory effect on the progression of myopia, and OK is now receiving increased attention and becoming popular as a treatment modality for myopic children [3–6].

Although this technique is well established in clinical practice, limited number of studies have investigated the underlying changes in corneal tissue. In previous studies, Swarbrick et al. found central epithelial thinning by 15.8 μm and mid-peripheral stromal thickening by 7.9 μm after 1 month of OK lens wear using an optical pachymeter [2, 7]. Choo et al. reported central epithelial thinning and mid-peripheral thickening in their cat model histologic study [8]. Nieto-Bona et al. reported epithelial thinning by 13% in the central corneal and 2 mm around the center [9, 10]. Wang et al. reported central epithelial thinning by 5.1% and mid-peripheral epithelial thickening by 1.9% on the temporal side and by 2.4% on the nasal side after one night of OK wear using a time-domain optical coherence tomography (OCT) [11]. However, Alharbi and Swarbrick found no significant thickening of the epithelium in the mid-periphery [7]. More recently, Lian et al. reported thinning of the central epithelium by 14–16% after seven nights of wear, while the horizontal mid-peripheral epithelium became thicker by approximately 6% and the superior epithelium became thinner [12]. However, all previous studies have only measured the changes in corneal apex and specific locations along the horizontal or vertical meridian. Overall, previous studies have concluded that refractive changes after OK lens wear are primarily due to central epithelial thinning, but change in mid-peripheral area and stromal remodeling profiles with its role in refractive change have not been clearly addressed [13].

The recent availability of corneal epithelial imaging by RTVue OCT with a corneal anterior module (Optovue, Inc., Fremont, CA, United States) provides a practical tool for clinical in vivo epithelial mapping, which demonstrates good repeatability in normal, keratoconus, post-LASIK (laser assisted in situ keratomileusis), and post-SMILE (small incision lenticule extraction) eyes [14–17]. This topographic thickness mapping shows average thickness values on 17 defined zones over a 6 mm diameter, which encompasses entire central flattening and part of mid-peripheral steepening zones. Therefore, it is suitable for recording topographic profiles of pachymetry changes and determining differential epithelial and stromal changes that may occur during OK treatment.

In this study, we investigated changes in epithelial and stromal thickness in subjects whose duration of OK treatment ranged from 2 weeks to 10 months. In addition, we investigated the association between corneal epithelial and stromal thickness changes and refractive changes.

## Materials and methods

### Patients

This study included a retrospective cohort of 60 eyes of 36 subjects that had been successfully fitted with OK lenses to treat myopia. All subjects had myopia of no more than -5.00 diopters (D) and astigmatism of no more than -1.50 D. The spherical refractive error at baseline was -2.37 ± 1.04 D (range: -1.00 to -5.00 D). A complete ophthalmic examination was performed to screen for corneal abnormalities and determine patient eligibility for OK. Standard subjective refraction was used to determine the manifest refractive error at baseline and subsequent visits. The inclusion criteria were as follows: age 7 to 25 years, stable manifest refraction with an unaided distance visual acuity of 20/25 or better after OK treatment, and the subject exhibiting a relatively well-centered target ring (bull’s eye pattern) in corneal topography after OK treatment. The exclusion criteria were as follows: a history of using hard contact lenses and the presence of any ocular pathologic conditions. This study protocol was approved by the Institutional Review Board of our institute and conducted according to the tenets of the Helsinki Declaration. Written informed consent to participate in this research was obtained from each patient.

### OK lens fitting

OK lenses from Paragon CRT (Paragon Vision Sciences, Mesa, AZ, United States) were used for all subjects. The following procedure was implemented by a single experienced ophthalmologist (W Kim) according to the manufacturer’s instructions: (1) the specifications for the lens were determined using the calculation ruler provided by the manufacturer; (2) adequate fit was assessed using fluorescein; and (3) a satisfactory fit was confirmed by the typical bull’s eye pattern in the corneal topography after an overnight trial. At each visit after OK lens wear, a complete contact lens follow-up examination was performed, including slit lamp, visual acuity, subjective refraction, keratometry (K), and corneal topography (Oculus Inc., Arlington, WA, United States), to monitor the response to OK lens wear. A separate evaluator reviewed medical records and corneal topography (B Kim), and this study included only subjects with satisfactory clinical outcomes.

### Measurement of corneal epithelial and stromal thickness

The corneal epithelial and total thickness data was obtained at baseline examination and during the follow-up visit when a stable refraction with an unaided distant visual acuity of 20/25 or better and satisfactory topographic change were achieved. Using the RTVue OCT system with a corneal anterior module (CAM) set at a wavelength of 830nm (Optovue, Inc., Fremont, CA, United States), we scanned the cornea in 8 meridians by using a “Pachymetry + Cpwr” scan (software version A6.11.0.12) over a 6 mm diameter centered at the corneal vertex. The corneal epithelial thickness (CET) maps were generated using an automatic algorithm and were divided into a total of 17 sectors: a central 2 mm diameter zone, eight paracentral zones within an annulus between the 2 and 5 mm diameter rings, and eight mid-peripheral zones within an annulus between the 5 and 6 mm diameter rings. In addition, thickness of 8 locations at 6 mm ring were recorded as cursor moved along the 6 mm border. We generated stromal thickness maps by subtracting the epithelial thickness from the corneal thickness as described previously [17, 18]. The mean values of 8 measurements in the paracentral zones, mid-peripheral zones, and 6.0 mm ring were calculated for statistical analysis. The thickness values for the left eyes were mirrored onto the right eye values so that the nasal/temporal characteristics could be maintained. The same investigator conducted all OCT imaging, and two consecutive images with SSI (signal strength index) > 30 were obtained to ensure the validity of the data, and the average value was used for analysis. All OCT pachymetry measurements were performed 8 hours after lens removal to minimize the potential influence of cornea edema or diurnal variation in corneal thickness [7, 19].

### Statistical analysis

**Fig 1:**
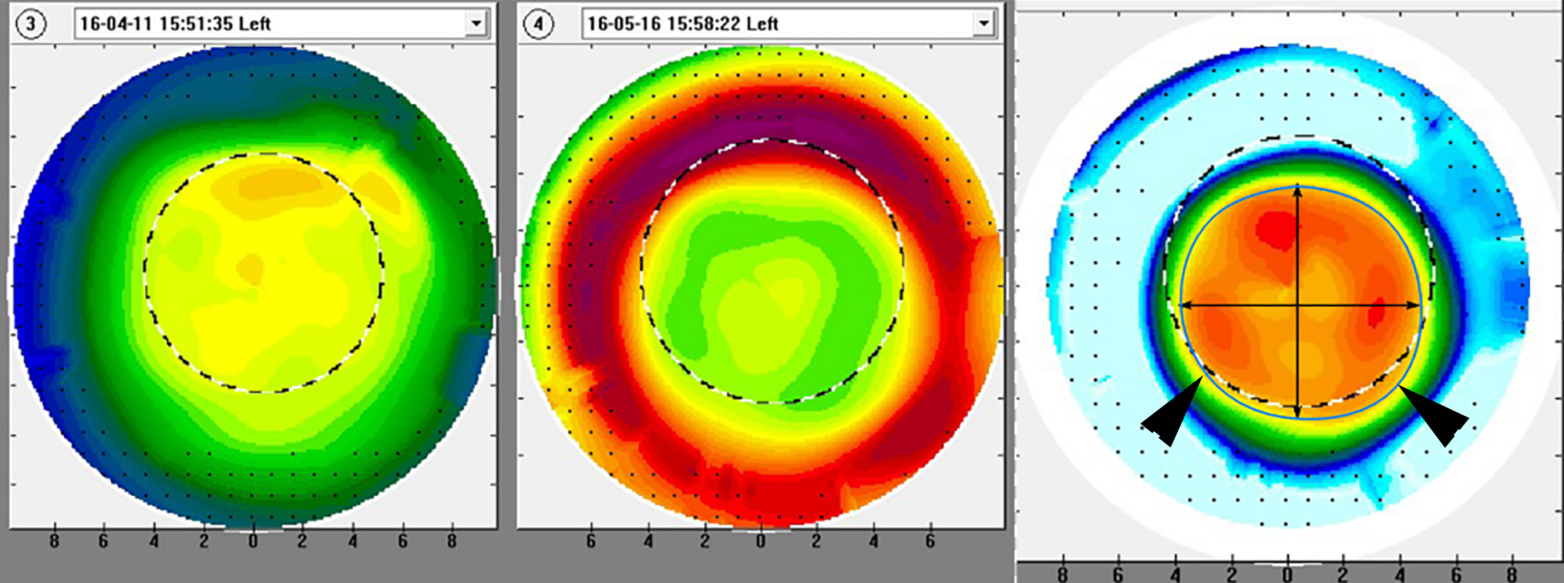
Measurement of treatment zone size. Treatment zone was derived from differential tangential map between baseline and post-OK fitting, which was equivalent to central flattening area surrounded by mid-peripheral annular ring of corneal steepening. Horizontal and vertical diameters of treatment zone were indicated by the lines with arrow heads, and treatment zone was demarcated by the blue circle of best fit indicated by the larger arrow head symbols. Treatment zone diameter and area were measured using pixel length and area from Image J software, which was converted to the mm using the scale provided by in the corneal topography.

Statistical analyses were performed using SPSS version 21.0 for Windows (IBM Corp, Armonk, NY, United States). All thickness and topographic variables in each group are confirmed for normality using Shapiro-Wilk test. Changes in refractive and topographic variables, as well as corneal epithelial and stromal thickness values, were compared using the paired t-test before and after OK lens wear. Subjects were subdivided into 3 groups according to the duration of OK lens treatment (group 1: 2 to 4 weeks; group 2: 4 to 12 weeks; group 3: >12 weeks) to investigate any difference in the magnitude of changes in refractive error and corneal thickness. One-way analysis of variance (ANOVA) was used to compare differences among these 3 subgroups. To model the expected change in refractive error based on baseline measurements and measured changes in the corneal thickness, we modified a formula presented by Munnerlyn et al. This formula has been used to determine ablation depth required in photorefractive keratectomy to achieve a given refractive change over a defined ablation zone diameter, and it is expressed as *T = -S*^2^ X *D*/8(*n-1*), where *T* is ablation depth, *S* is ablation diameter (both in meters), *D* is the desired manifest refractive change in diopters, and n is the refractive index of the cornea (1.377) [20]. Instead of using sagittal height change as suggested previously [1, 2], we substituted the change in CET for *T* and the treatment zone diameter for *S*, and compared the refractive change predicted by this formula with the observed refractive changes. In addition, simple linear regression analyses were performed to investigate the influence of baseline refraction, topographic parameters, epithelial and stromal thickness, changes in thicknesses, and treatment zone size. Treatment zone was defined as central flattening zone demarcated by the circle of best fit determined by points of zero corneal power change on the differential tangential map between baseline and post-OK fitting (Fig 1). Horizontal and vertical diameters and area of treatment zone were then measured using 8 adjustable points provided by Image J software (National Institutes of Health, Bethesda, MD, United States) and converted to mm using the scale provided by the corneal topography. All statistical significance was defined as P < 0.05.

## Results

### Topography and epithelial thickness changes

The study consisted of 60 eyes of 26 male and 34 female patients successfully treated with OK lenses for correction of myopia. Mean age of the subjects was 13.4 ± 6.7 years (range: 7 to 25 years), and all subjects were treated with overnight OK lenses for at least 2 weeks with a mean duration of 10.9 weeks (range: 2 to 40 weeks). Spherical equivalent refractive error (manifested refraction) decreased after treatment, as summarized in Table 1.

**Table 1:** Changes in refractive and topographic indices.

N = 60		Pre	Post	P value
**Refractive**				
Sphere (D)		-2.22±0.99	-0.34±0.52	**< 0.001**
Cylinder (D)		-0.29±0.30	-0.57±0.46	**< 0.001**
Spherical equivalent (D)		-2.37±1.04	-0.63±0.64	**< 0.001**
**Topographic**				
Flat K (D)		42.25±1.17	41.01±1.15	**< 0.001**
Steep K (D)		43.21±1.33	42.00±1.23	**< 0.001**
Astigmatism (D)		0.96±0.40	0.99±0.59	0.561
Eccentricity		0.56±0.10	-0.12±0.33	**< 0.001**

D; diopter. Data are presented mean ± standard deviation.

**Fig 2:**
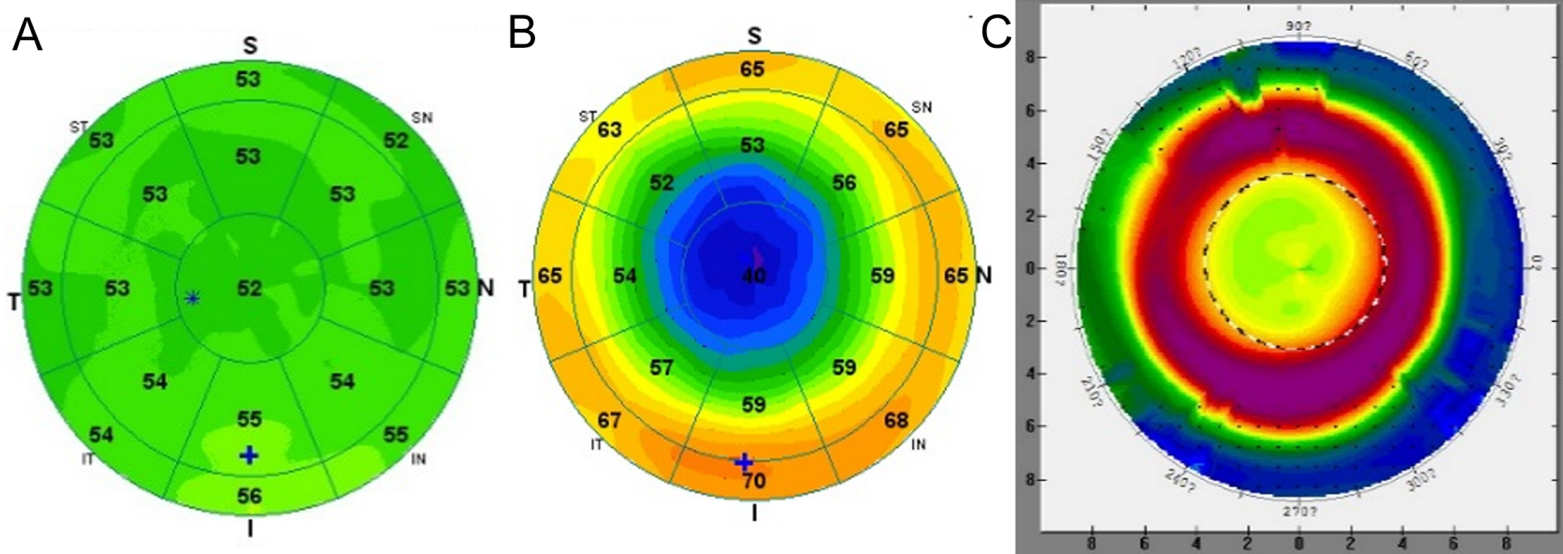
Representative corneal epithelial thickness and topography during orthokeratology. The corneal epithelial thickness maps are divided into a total of 17 sectors: a central 2 mm diameter zone, eight paracentral zones within an annulus between the 2 and 5 mm diameter rings, and eight mid-peripheral zones within an annulus between the 5 and 6 mm diameter rings. The epithelial thickness map shows central compression area surrounded by annular mid-peripheral thickening area and they seem to fit to tangential topographic images. (A) Baseline epithelial thickness, (B) post-OK epithelial thickness map and (C) post-OK tangential topography.

All subjects showed central epithelial compression after OK lens wear and overall shape of epithelial thickness map corresponded well with tangential topography. Our topography analysis indicated that the mean diameter of treatment zone was 3.55 ± 0.54 mm horizontally (range; 2.75–5.0 mm) and 3.41 ± 0.49 mm vertically (range: 2.5–4.75 mm). On average, there was a slight tendency of decentering of treatment zone toward temporal (0.25 ± 0.31 mm) and inferior (0.23 ± 0.40 mm) side. Fig 2 showed a representative epithelial thickness and topography maps.

Table 2 shows corneal thickness before and after OK lens wear. Overall, total corneal thickness decreased in the center and increased in the paracenter and mid-periphery. The CET decreased by 16% (8.4 ± 3.3 μm) at the center and increased by 5.1% (2.7 ± 3.7 μm) in the mid-periphery. When compared with baseline values, stromal thickness, however, increased less centrally but increased radially (centrifugally) toward the mid-periphery. The CET at 6.0 mm zone showed significant thickening compared to baseline values, but no significant differences were noted with changes in mid-periphery (5–6 mm annular ring).

**Table 2:** Corneal thickness changes before and after orthokeratology.

	Before	Range	After	Range	P value
**Total**					
Central (μm)	538 ±33.0	446–592	532±31.8	441–593	< 0.001
Paracentral (μm)	558 ±33.2	465–613	560±32.0	475–624	0.013
Mid-periphery (μm)	580±35.0	475–637	586±31.9	512–656	< 0.001
6.0 mm zone	584±35.9	471–642	589±31.9	517–653	0.017
**Epithelium**					
Central (μm)	52.6±2.72	47.0–59.0	44.1±4.14	34.0–53.0	< 0.001
Paracentral (μm)	52.9±2.40	46.4–58.6	51.4±3.28	44.0–59.1	< 0.001
Mid-periphery (μm)	53.0±2.27	46.8–57.5	55.7±4.18	43.1–66.8	< 0.001
6.0 mm zone	53.0±2.34	46.5–58.0	55.9±4.49	44.1–68.4	<0.001
**Stroma**					
Central (μm)	486±32.4	395–540	488±30.9	405–549	0.004
Paracentral (μm)	505±32.8	414–562	508±31.4	428–574	< 0.001
Mid-periphery (μm)	527±34.7	424–587	531±31.4	457–600	0.004
6.0 mm zone	531±35.6	420–592	533±31.9	461–599	0.418

Data are presented mean ± standard deviation.

**Fig 3:**
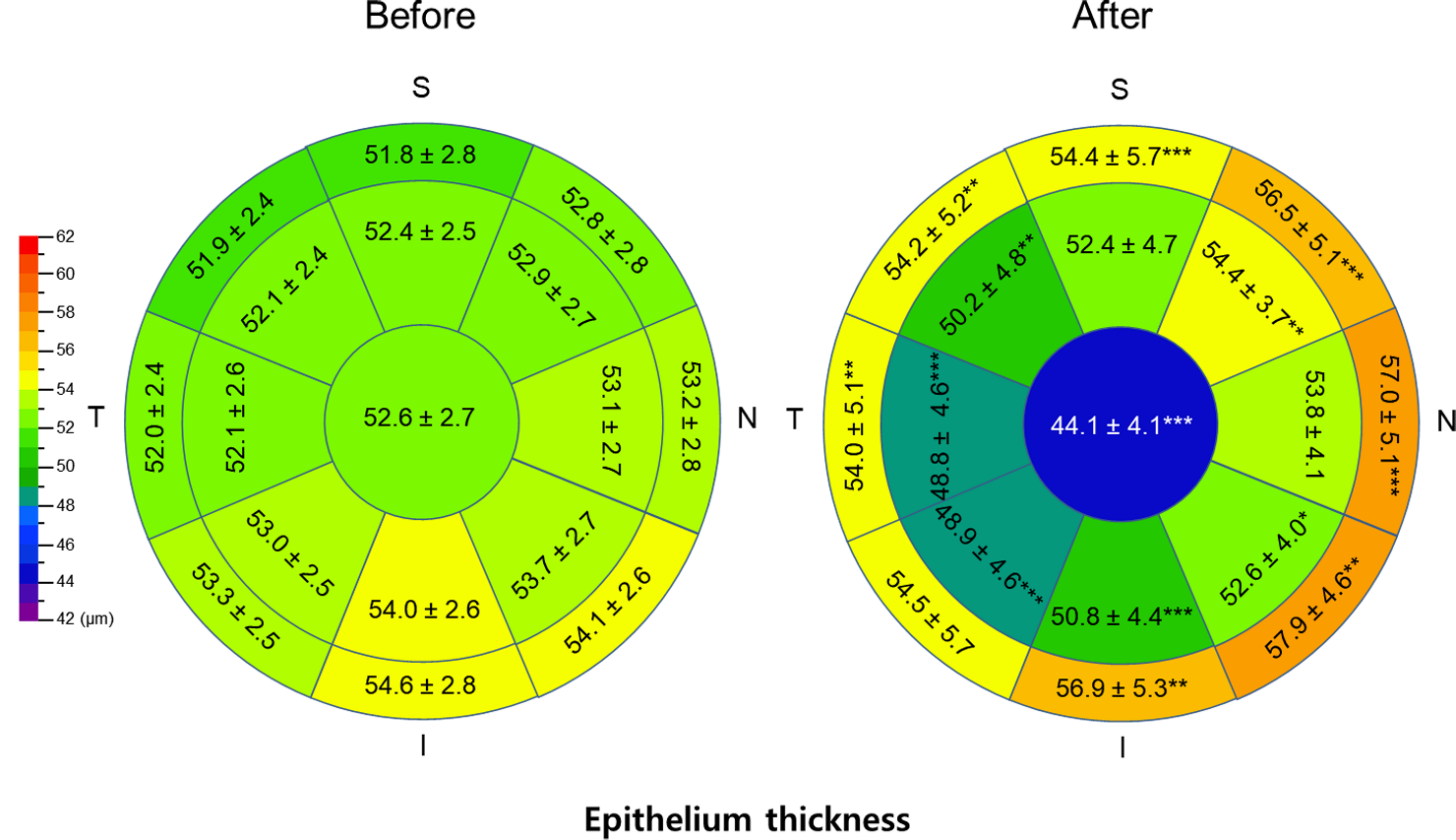
Topographic corneal epithelial thickness map before and after orthokeratology. The corneal epithelium thinned from the center to the paracenter, and the mid-peripheral epithelium thickened. There was more thinning of the temporal and inferior zones compared with the nasal and superior zones of the paracenter, with further thickening in the nasal zone compared with the temporal zone in the mid-periphery. *P<0.05, **P<0.01, and ***P<0.001.

Sectoral comparison of baseline epithelial thickness showed that the inferior epithelium is thicker than the superior one, and the nasal epithelium was thicker than the temporal one. Changes in epithelial thickness showed non-uniform pattern; more thinning of the temporal and inferior zones compared with the nasal and superior zones of the paracenter, with further thickening in the nasal zone compared with the temporal zone in the mid-periphery (Fig 3).

In the subgroup analysis for treatment duration, no demographic differences were found among the three groups (Table 3). There was also no difference in corneal thickness changes among the three groups. Although there is no statistical significance, a slight tendency toward further thinning was noted in subjects in the group wearing OK lenses for a longer duration.

**Table 3:** Corneal thickness change in 3 groups, divided by duration of orthokeratology.

	2 to 4 weeks	5 to 12 weeks	> 12 weeks	P value
Subjects (numbers)	26	16	18	
Using periods (weeks)	2.81±1.17	8.56±1.90	24.56±9.33	< 0.001
Age (years)	15.42±7.21	11.94±6.13	11.67±5.83	0.112
Sphere (change)	1.76±1.07	1.89±0.52	1.99±0.51	0.652
**Total**				
Central (μm)	-4.85±5.61	-6.06±5.21	-8.22±7.08	0.193
Paracentral (μm)	3.26±4.62	2.53±5.26	-0.47±6.51	0.080
Mid-periphery (μm)	6.79±9.01	8.92±10.34	4.68±8.53	0.417
**Epithelium**				
Central (μm)	-7.62±3.63	-8.19±2.95	-9.56±2.87	0.155
Paracentral (μm)	-0.60±2.53	-1.62±2.61	-2.34±1.75	0.057
Mid-periphery (μm)	3.43±4.22	1.83±3.32	2.13±2.89	0.311
**Stroma**				
Central (μm)	2.77±5.20	2.13±5.06	1.33±5.77	0.682
Paracentral (μm)	3.85±5.01	4.15±6.45	1.87±5.54	0.411
Mid-periphery (μm)	3.36±9.27	7.09±11.19	2.55±7.38	0.323

### Correlation analysis

Simple linear regression analysis showed that there was no association between baseline cornea thickness and the magnitude of refractive change. The magnitude of change in CET was positively associated with a change in paracentral epithelial thickness and negatively associated with changes in stromal thicknesses of the mid-periphery. The decrease in CET showed a significant linear association with the magnitude of refractive error correction. Stromal thickening in the center, paracenter, and mid-periphery showed significant associations with each other; however, no significant association was observed with refractive correction (Table 4).

**Table 4:** Simple regression between myopia correction and various factors.

	Change in refractive error (D)			
	R^2^	B	β	P
**Age**	-0.017	-0.003	-0.022	0.868
**Lens factor**				
Base curve (mm)	0.283	0.135	0.544	**< 0.001**
Delta K (base curve-flat K, D)	0.743	-0.620	-0.865	**< 0.001**
Treatment zone vertical diameter (mm)	0.037	-0.361	-0.232	0.075
Treatment zone horizontal diameter (mm)	0.101	-0.495	-0.340	**0.008**
Treatment zone area (mm^2^)	0.087	-0.022	-0.320	**0.013**
Duration of OK treatment (weeks)	-0.009	0.007	0.090	0.492
**Topographic variables**				
Flat K (before, D)	0.014	0.120	0.175	0.181
Change in flat K (D)	0.136	-0.613	-0.388	**0.002**
Steep K (before, D)	0.038	0.140	0.233	0.073
Change in steep K (D)	0.036	-0.272	-0.228	0.080
Eccentricity (before)	0.028	1.657	0.211	0.105
Eccentricity (change)	0.034	-0.514	-0.225	0.083
**Change in thickness**				
**Epithelium**				
Central (μm)	0.390	-0.152	-0.633	**< 0.001**
Paracentral (μm)	0.060	-0.090	-0.276	0.053
Mid-periphery (μm)	-0.015	0.009	0.043	0.743
**Stroma**				
Central (μm)	0.043	-0.037	-0.244	0.060
Paracentral (μm)	-0.012	-0.010	-0.069	0.601
Mid-periphery (μm)	0.008	0.013	0.157	0.231
**Total**				
Central (μm)	0.297	-0.073	-0.556	**< 0.001**
Paracentral (μm)	0.020	-0.028	-0.191	0.143
Mid-periphery (μm)	0.015	0.015	0.178	0.174

**Fig 4:**
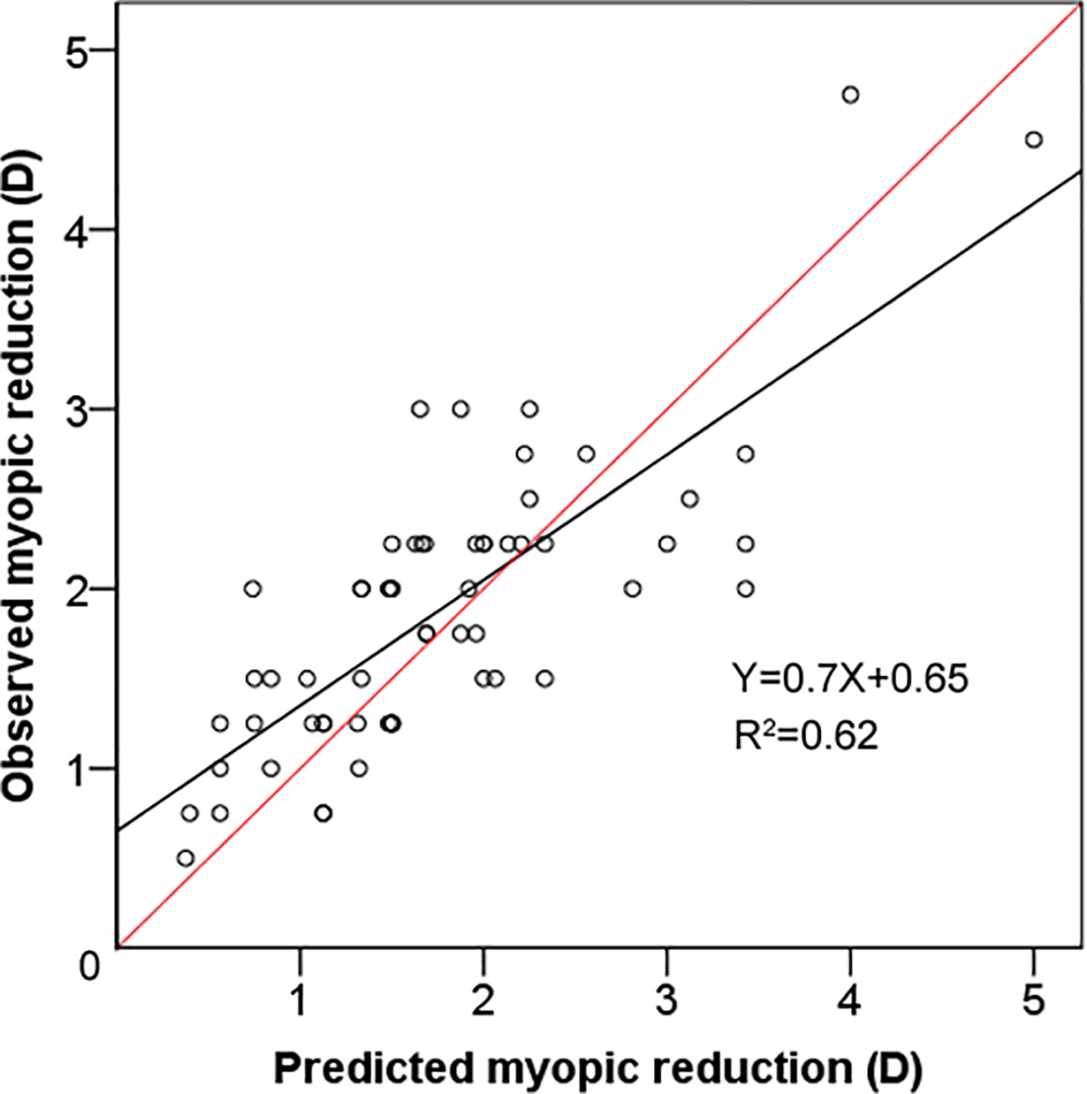
Relationship between changes in refractive error predicted by Munnerlyn’s formula and refractive changes observed after orthokeratology. Changes in central epithelial thickness and treatment zone diameter are substituted for ablation depth and diameter, respectively in Munnerlyn’s formula. The red line represents equality between the measured refractive change and the predicted refractive change.

A flatter base curve and a larger difference between baseline K and the base curve are associated with increased change in refractive error. There was an inverse correlation between the magnitude of refractive correction and the treatment zone size (horizontal diameter and area). Munnerlyn’s formula could also predict the observed refractive change after substituting change in measured epithelial thickness for ablation depth and the measured treatment diameter for ablation diameter (Fig 4).

## Discussion

Using SD-OCT, we were able to investigate topographic changes in epithelial, stromal, and total corneal thicknesses before and during OK treatment, and we demonstrated that refractive changes were due to central epithelial thickness changes. Previous studies have observed central epithelial thinning using various methods, including optical pachymeter [11, 12], confocal microscopy [9, 10], and OCT [11, 12], and it is now a well-known characteristic of OK. Changes in the mid-peripheral area and in stromal thickness, however, have not been thoroughly studied; their associations with the change in refractive error need to be further investigated. As opposed to the central epithelium, there are some conflicts in the remodeling pattern in the mid-periphery zone. Alharbi and Swarbrick [7] found only stromal thickening, whereas other groups [11] found significant epithelial thickening in the horizontal meridian. Our study revealed thickening not only in the horizontal meridian, but also in the superior and inferior zone, which was in disagreement with the study of Lian et al [12]. The different methods or different measurement locations used in previous studies might explain these differences. Our overall topographic thickness change map showed more epithelial compression in temporal paracenter than in the nasal zone and more thickening in the nasal mid-periphery than in the temporal zone. This seems partly related to the centering of the OK lenses. Our result indicated a tendency of decentering of treatment zone toward temporal (0.25 ± 0.31 mm) and inferior (0.23 ± 0.40 mm) side, which is in good agreements with previous studies [21, 22]. On the other hand, the asymmetric compression pattern can be attributable to inherent OK effect on asymmetric corneal surface [23]. Of note, asymmetry of epithelial thickness between nasal and temporal sectors increased after OK treatment and it was greater than that of vertical meridian (Fig 3). This finding is consistent with a previous research reporting a greater flattening in the temporal than nasal sector [23].

The current study showed significant thickening of both the epithelium and stroma in the mid-peripheral zone (5 to 6 mm) that nearly coincides with tear film pooling zone caused by the reverse curve zone of the OK lens. Our study also demonstrates that thickening of neither the epithelium nor stroma in the mid-peripheral zone was associated with the observed refractive change. A possible explanation of no associations between refractive change and changes in paracentral or mid-peripheral thickness comes from the limitation of our device in imaging area and sectoral analysis method. In typical subjects with 3–4 mm central compression area, the mid-peripheral annular steepening was located at 4–6 mm (Fig 2). There must be averaging effects of compression and steepening in paracentral sectors in some subjects with smaller treatment zone and maximal corneal steepening area would not always be in 5–6 mm annular ring. On the other hand, maximal steepening area may not be imaged in some cases with larger treatment zone, which could lead to an underestimation. We believe that thickness changes in 5–6 mm annular ring and 6 mm ring provided an average effect in subjects with 3–4 mm central compression area. However, a discrepancy between measurement area and maximal response area would underestimate mid-peripheral thickness change. For future analysis, an individualized localization with wider imaging area should be warranted to fully address the possible association between mid-peripheral thickness change and refractive change.

The nature of epithelial and stromal thickening is not clear. The stromal edema could be hypothesized, but previous researches did not attribute this stromal thickening to overnight edema response.[7, 19] Alharbi et al. reported inhibition in central stromal edema response, as the positive pressure behind the lens might exert a “clamping effect” on the central stoma. The researchers also reported that, although central and peripheral corneal stromal edema showed complete daytime recovery to baseline, gradual residual stromal thickening remained in the stromal mid-periphery with continued overnight lens wear [19]. Our result showed that stromal thickening was found less in center than in mid-periphery, which is consistent with the previous study. Of note, we found significant associations among stromal thickness changes in the center, paracenter, and mid-periphery. Although central stromal thickening was less prominent, it was proportional to thickening of other areas. From this point of view, we assume that stromal thickness remodeling is, to some extent, affected by the same factors, such as negative pressure behind the reverse curve zone. The nature of epithelial thickening in the mid-periphery is also unclear. It is neither associated with refractive change nor with change in CET. It may be caused by epithelial deformation caused by negative pressure exerted by the reverse curve zone. Epithelial redistribution from the center could lead to thickening in this area [9, 10]. We hypothesize that central compression may hinder epithelial migration from the limbus, and migrated cells may therefore redirect or accumulate in the mid-periphery. Further work is necessary to elucidate the nature of the cellular events underlying the stromal and epithelial thickness changes found in this study.

Another interesting finding of this study concerns factors affecting refractive change. As mentioned earlier, the amount of refractive change was proportional to the magnitude of epithelial thinning and inversely associated with treatment zone diameter. This association prompted us to fit Munnerlyn’s formula to the measured refractive change. Swarbrick et al. previously used this formula to model the expected change in refractive error based on measured changes in corneal thickness [2, 7]. Instead of using sagittal height change as suggested by Swarbrick, we substituted change in CET for ablation depth and the measured treatment zone diameters for ablation diameter from Munnerlyn’s formula and compared the refractive change predicted by this formula with the observed refractive changes (Fig 4). This model closely predicts the measured refractive changes found in this subject group (*R*^2^ = 0.62; *P <* 0.001). Our result reconfirms previous findings that there is a linear relationship between the degree of myopic correction and the magnitude of CET thinning, and an inverse relationship with the treatment zone diameter. In addition, stromal thickening shows no correlation with refractive change.

Possible limitations of this study are the cross-sectional design and different follow-up periods. A previous meta-analysis of central corneal thickness (CCT) change following OK concluded that CCT reduction mostly occurred within 1 week and remained stable for 1 month [13]. The current study did not focus on the timing of the thickness change. Rather, we were more interested in consistent change after obtaining refractive stability. As for the CCT change over 3 months, this has not been documented by many studies. Although the current study could not provide chronological changes, we compared the magnitude of change in epithelial and stromal thickness between subgroups with different treatment durations. We found no significant further compression or thickening between subgroups.

In this study, we presented topographic thickness changes in corneal epithelium and stroma using epithelial thickness map. Since orthokeratology possibly compromises epithelial integrity by inducing epithelial thinning, monitoring corneal epithelium is of paramount importance [24]. We believe that the epithelial thickness map is useful as it provides indicators of corneal health such as epithelial irregularity, erosion or edema. It is also as good as topography for monitoring compression effect (response of OK effect), centration, treatment zone size, etc.

In conclusion, this study demonstrates that orthokeratology causes the central corneal epithelium to thin while the mid-peripheral epithelium and stroma become thicker. Refractive changes induced by overnight OK can be explained by the induced changes in CET, rather than by overall bending of the corneal tissue. Overall, OK is a safe option for temporary myopia correction as long as recommended care regimen and follow-up is adherent to. Monitoring epithelial health will assure timely and appropriate treatment for complication.
